# Stability Evaluation of Brain Changes in Parkinson's Disease Based on Machine Learning

**DOI:** 10.3389/fncom.2021.735991

**Published:** 2021-10-26

**Authors:** Chenggang Song, Weidong Zhao, Hong Jiang, Xiaoju Liu, Yumei Duan, Xiaodong Yu, Xi Yu, Jian Zhang, Jingyue Kui, Chang Liu, Yiqian Tang

**Affiliations:** ^1^School of Life Science and Technology, University of Electronic Science and Technology of China, Chengdu, China; ^2^Center for Information in Medicine, University of Electronic Science and Technology of China, Chengdu, China; ^3^MOE Key Lab for Neuroinformation, The Clinical Hospital of Chengdu Brain Science Institute, Chengdu, China; ^4^College of Computer, Chengdu University, Chengdu, China; ^5^Department of Neurosurgery, Rui-Jin Hospital, Shanghai Jiao Tong University School of Medicine, Shanghai, China; ^6^Department of Abdominal Oncology, Cancer Center, West China Hospital, Sichuan University, Chengdu, China; ^7^Department of Computer and Software, Chengdu Jincheng College, Chengdu, China; ^8^School of Physics and Electronic Engineering, Sichuan Normal University, Chengdu, China; ^9^Department of Urology, Tonghai County People's Hospital, Yuxi, China

**Keywords:** ReliefF, graph theory, RFE, stability selection, machine learning, Parkinson's disease, magnetic resonance imaging

## Abstract

Structural MRI (sMRI) has been widely used to examine the cerebral changes that occur in Parkinson's disease (PD). However, previous studies have aimed for brain changes at the group level rather than at the individual level. Additionally, previous studies have been inconsistent regarding the changes they identified. It is difficult to identify which brain regions are the true biomarkers of PD. To overcome these two issues, we employed four different feature selection methods [ReliefF, graph-theory, recursive feature elimination (RFE), and stability selection] to obtain a minimal set of relevant features and nonredundant features from gray matter (GM) and white matter (WM). Then, a support vector machine (SVM) was utilized to learn decision models from selected features. Based on machine learning technique, this study has not only extended group level statistical analysis with identifying group difference to individual level with predicting patients with PD from healthy controls (HCs), but also identified most informative brain regions with feature selection methods. Furthermore, we conducted horizontal and vertical analyses to investigate the stability of the identified brain regions. On the one hand, we compared the brain changes found by different feature selection methods and considered these brain regions found by feature selection methods commonly as the potential biomarkers related to PD. On the other hand, we compared these brain changes with previous findings reported by conventional statistical analysis to evaluate their stability. Our experiments have demonstrated that the proposed machine learning techniques achieve satisfactory and robust classification performance. The highest classification performance was 92.24% (specificity), 92.42% (sensitivity), 89.58% (accuracy), and 89.77% (AUC) for GM and 71.93% (specificity), 74.87% (sensitivity), 71.18% (accuracy), and 71.82% (AUC) for WM. Moreover, most brain regions identified by machine learning were consistent with previous findings, which means that these brain regions are related to the pathological brain changes characteristic of PD and can be regarded as potential biomarkers of PD. Besides, we also found the brain abnormality of superior frontal gyrus (dorsolateral, SFGdor) and lingual gyrus (LING), which have been confirmed in other studies of PD. This further demonstrates that machine learning models are beneficial for clinicians as a decision support system in diagnosing PD.

## 1. Introduction

Parkinson's disease (PD), a serious neurodegenerative disease, is caused by the deterioration of dopaminergic neurons (Group, 2002; Perez-Lloret and Rascol, 2010). It has been reported that disrupted dopamine transmission leads to abnormal motor symptoms and beta oscillations in the subthalamic nucleus (STN) in patient with PD (Mallet et al., 2008). Moreover, the lack of dopamine can damage several areas of the brain, producing a variety of motor and nonmotor symptoms such as resting tremor, bradykinesia, muscle rigidity, depression, and sleep disorders (Koller et al., 1989; Morris, 2000; Chaudhuri and Schapira, 2009; Fox et al., 2011). This disease affects millions of people worldwide and reduces quality of life and happiness. Therefore, the early diagnosis and treatment of PD are particularly important. In general, physicians identify the severity/stage/progression of PD by medical history and neurological examination (Folstein et al., 1975; Fahn et al., 1997). However, the results of these clinical examinations are heavily affected by the knowledge and experience of clinicians, posing a risk to the accurate diagnosis and effective treatment of PD (Fahn et al., 1997; MD et al., 2006; Jankovic, 2008). In order to better diagnose and treat PD, computer-based data analysis methods have been gradually applied to the imaging of neurodegenerative diseases (Hirschauer et al., 2015; Peker et al., 2015; Rana et al., 2015b). The microstructure of gray matter (GM) and white matter (WM) in the brains of patients with PD may change at an early stage of the disease, and structural changes occur earlier than physiological changes (Rektor et al., 2018). Existing studies have shown that neuroimaging analyses can reflect changes in brain microstructure. The development of MRI has made it possible to study the structure and function of the human brain in a noninvasive manner. Structural MRI (sMRI) (Heim et al., 2017) is widely used in the study of neuroimaging due to its advantages of good contrast and high resolution (Duchesne et al., 2009; Ziegler and Augustinack, 2013).

Most existing research studies based on sMRI have used conventional statistical analysis methods to discriminate patients with PD from healthy subjects. It has been reported that patients with PD show GM reductions compared with healthy controls in the bilateral temporal lobe, bilateral occipital lobe, bilateral parietal lobe, bilateral frontal lobe, bilateral insular lobe, bilateral parahippocampal gyrus, bilateral amygdala, right uncus, right precuneus, caudate, and right posterior lobe of the cerebellum (Price et al., 2004; Summerfield et al., 2005; Agosta et al., 2013; Xia et al., 2013; Moccia et al., 2016; Gao et al., 2017; Kikuchi et al., 2017). Price et al. (2004), Agosta et al. (2013), and Moccia et al. (2016) analyzed WM and observed brain changes in the regions of the middle temporal gyrus, right superior longitudinal fasciculus/angular gyrus, and insula. Schwarz et al. (2011) observed changes in the substantia nigra based on manually predefined region(s)-of-interest (ROI(s)). However, conventional statistical analysis often overlooks the correlations among voxels and focuses on group-level differences rather than individual-level diagnosis.

In order to address these issues, machine learning methods have been increasingly applied to neuroimaging in recent years (ChenZhiHong et al., 2020; Liu et al., 2020). Machine learning methods have been widely used for classification studies aiming to distinguish between PD and HC controls (Duchesne et al., 2009; Long et al., 2012; Lei et al., 2018). In addition to being sensitive to subtle differences in the brain, machine learning methods can also be generalized to the diagnosis of individual patients. In neuroimaging studies, the number of features (voxels) is often much larger than the number of subjects, which is a very common problem in machine learning, called the “curse of dimensionality” (Wang et al., 2017, 2019; Altman and Krzywinski, 2018). This problem can easily lead to the overfitting of machine learning models (Guyon and Elisseeff, 2003). Hence, it is essential to utilize feature selection to capture the most relevant features and remove redundant ones.

In general, feature selection includes supervised feature selection and unsupervised feature selection. According to the different attributes of the features, supervised feature selection methods can be divided into three categories: (1) filter methods (Biesiada and Duch, 2005; Sánchezmaroño et al., 2007), which are based on simple statistical parameters (mean value, variation and correlation coefficient, etc.) and rank them in terms of their ability to detect group-level differences; (2) wrapper methods, which are based on the cost function, and sort all features based on their degree of correlation (Kohavi and John, 1997; ChenZhiHong et al., 2020); and (3) embedded methods (Wang et al., 2015), which select relevant features by imposing certain “penalties” to obtain a subset of relevant features. Filtering methods have the benefit of low computational cost, while wrapper methods are superior to filtering methods in performance due to their discriminative ability (Lee and Verleysen, 2007; Chu et al., 2011; Adeli et al., 2016; Cigdem et al., 2018). Considering the interaction among features, embedded methods have shown excellent performance in pattern classification research (Wang et al., 2015). Unsupervised feature selection methods construct related features by means of linear or nonlinear combinations of the original prediction features (Lee and Verleysen, 2007).

An increasing number of feature selection methods are being applied in the field of neuroscience. Chu et al. (2011) used a variety of different feature extraction techniques to construct a classification model, and experiments have revealed that feature selection methods can enhance the accuracy of classification. Cigdem et al. (2018) classified PD and controls using a probability distribution function based on feature selection methods to build separate decision models for GM and WM, which achieved good classification accuracy of 75.00 and 72.50%, respectively. However, the histogram technology used in that study leads to the loss of information. Adeli et al. (2016) also utilized an unsupervised joint feature-sample selection (JFSS) method to select an optimal subset of samples and features and construct a reliable diagnosis model, which achieved a classification accuracy of 81.9%. Rana et al. (2015a) utilized the filtering feature selection method based on mutual information in conjunction with a support vector machine (SVM) to construct the classification model, and the classification accuracy reached 86.67%. However, these studies were based on region-of-interest (ROI) analysis, which probably neglected some potential brain changes. Rana et al. (2015b) proposed an unsupervised feature selection method based on spectral graph theory and ultimately achieved a classification accuracy of 86.67%. Recently, Babu et al. (2014) employed a meta-cognitive radial basis function network (McRBFN) with recursive feature elimination (RFE) to construct a classification model, which achieved a classification accuracy of 87.21%.

Previous studies have sought to identify brain changes in PD based on voxel-based morphometry (VBM), which has provided the analysis at the level of group to understand the neurobiology of disease, while it has focused on the disease diagnosis of individual patients in a clinical context. Group-level analysis based on statistical analysis fails to implement individual diagnosis, because it only detects brain differences between groups. Furthermore, group-level analysis is sensitive to the acquisition parameters of the sMRI data (Marquand et al., 2013; Salvatore et al., 2013; Cherubini et al., 2014; Rana et al., 2015a,b). Due to the robustness of machine learning techniques, four popular feature selection methods (ReliefF, graph theory, RFE, and stability selection) (Mwangi et al., 2013; Tohka et al., 2016) were used to detect the morphological brain differences characterizing PD on structural brain MRI in our study. These feature selection methods were used to retrieve the minimum sets of relevant and nonredundant features from GM and WM separately. Then, we used SVM to learn the decision model from the selected features. Moreover, due to the excellent properties of the feature selection method, we were able to identify the most discriminative brain regions, and we evaluated their stability both horizontally and vertically. First, we compared the brain changes using different feature selection methods. Then, we conducted a vertical comparison of the brain changes found by machine learning with the results of previously reported traditional statistical analyses. The experimental results showed that machine learning techniques have superior and robust classification performance in distinguishing patients with PD from HCs, and most of the identified brain regions are consistent with previous findings, indicating that they were stable biomarkers of PD. The remaining sections of the present study are organized as follows: Section 2 briefly describes the materials. Section 3 gives a brief description including details on the preprocessing, feature selection, SVM classification, and performance evaluation metrics. Section 4 contains the experimental results and discussion, and section 5 presents the conclusion.

## 2. Materials

### 2.1. Subjects

All data carried in this study came from the Parkinson's Progression Markers Initiative (PPMI) datasets (https://www.ppmi-info.org/data) (Marek et al., 2011). Briefly, PPMI is a public repository from various centers that provides neuroimaging and associated clinical information of various modes of PD and matched control subjects for data sharing and scientific research. The PPMI cohort includes 600 datasets that comprise 400 participants with PD and 200 healthy subjects. All participants in PPMI have received approval from the Institutional Review Board (IRB). Inclusion criteria of patients with PD in the ppmi datasets were the following: (1) 30 years of age or older. (2) Patients had at least two of the following signs: resting tremor; rigidity; and slowness of movements, with at least one observation of bradykinesia (slowness). (3) Hoehn and Yahr stage 1 or 2 at baseline. Inclusion and exclusion criteria for healthy controls were as following: (1) 30 years of age or older. (2) Healthy subjects whose first-degree relatives had idiopathic PD were excluded. (3) Healthy subjects with a history of neurological disorders, mental illness, head injuries, or substance abuse were excluded.

In this study, we use the MRI data acquired by the PPMI study, in which a T1-weighted, 3D sequence is acquired for each subject using 3T SIEMENS scanners. We have considered 208 subjects (112 healthy subjects and 127 patients with PD) available in the datasets as of September 2019. Among 208 subjects, 18 healthy subjects and 21 patients with PD MR images were excluded due to failure of the segmentation method. Furthermore, all subjects were selected based on the following criteria: (1) All subjects were aged over 50 years and under 80 years; 14 healthy subjects and 17 patients with PD were excluded. (2) All subjects did not have depression, with a Geriatric Depression Scale score of < 5; 8 healthy subjects and 12 patients with PD were excluded. (3) All participant subjects were right-handed; 10 healthy subjects and 9 patients with PD were excluded. (4) patients with PD with Hoehn and Yahr stage < 3 were retained; 8 patients with PD were excluded. (5) The disease duration of patients with PD is greater than 12 months and less than 48 months; 15 patients with PD were excluded. Finally, 44 gender- and age-matched healthy subjects with a similar level of education were selected for comparison.

The disease severity and functional status of each patient were assessed with the Movement Disorder Society–Unified Parkinson's Disease Rating Scale-part III (MDS-UPDRS III) and Hoehn–Yahr (HY) stage. All subjects were assessed with the Montreal Cognitive Assessment (MoCA). The disease duration of patients with PD was defined from the time of symptom onset. The demographic details of the patients whose data were used in our study are shown in Table 1.

**Table 1:** Demographic information of data used in our study.

**Variable**	**PD**	**HC**	***p*-values**
Gender (M/F)	29/15	27/17	0.658^b^^*^
Age (years)	63.95 ± 8.13	63.89 ± 7.61	0.968^a^^*^
Education (years)	15.61 ± 2.88	16.07 ± 3.08	0.476^a^^*^
Disease duration (years)	2.81 ± 1.24	–	–
MDS-UPDRS III score	32.14 ± 9.85	–	–
H&Y score	2.05 ± 0.47	–	–
MoCA score	27.66 ± 2.49	28.23 ± 1.49	0.197^a^^*^

*PD, Parkinson's disease; HC, Healthy controls; M/F, male/female*.

*MDS-UPDRS III, Movement Disorder Society-Unified Parkinson's*.

*Disease Rating Scale-part III*.

*MoCA, Montreal Cognitive Assessment. H&Y = Hoehn-Yahr staging*.

a *Two-sample t-test*.

b *Chi-square test*.

* *Statistical analyses have not shown significant difference between healthy controls and PD according to age, sex, MoCA score, and education year*.

### 2.2. MRI Acquisition

In this study, we used a 3T SIEMENS scanner to obtain T1-weighted 3D sequences for each subject. T1-weighted imaging was acquired with the following parameters: acquisition plane=sagittal, acquisition type = 3D, coil = body, flip angle = 9.0 degrees, matrix X/Y/Z = 240.0/256/176 pixel, manufacturing model = TrioTim, pixel spacing X/Y = 1.0/1.0 mm, pulse sequence = GR/IR, slice thickness = 1 mm, and TE/TI/TR = 2.98/900/2300 ms.

## 3. Machine Learning Methods

**Figure 1:**
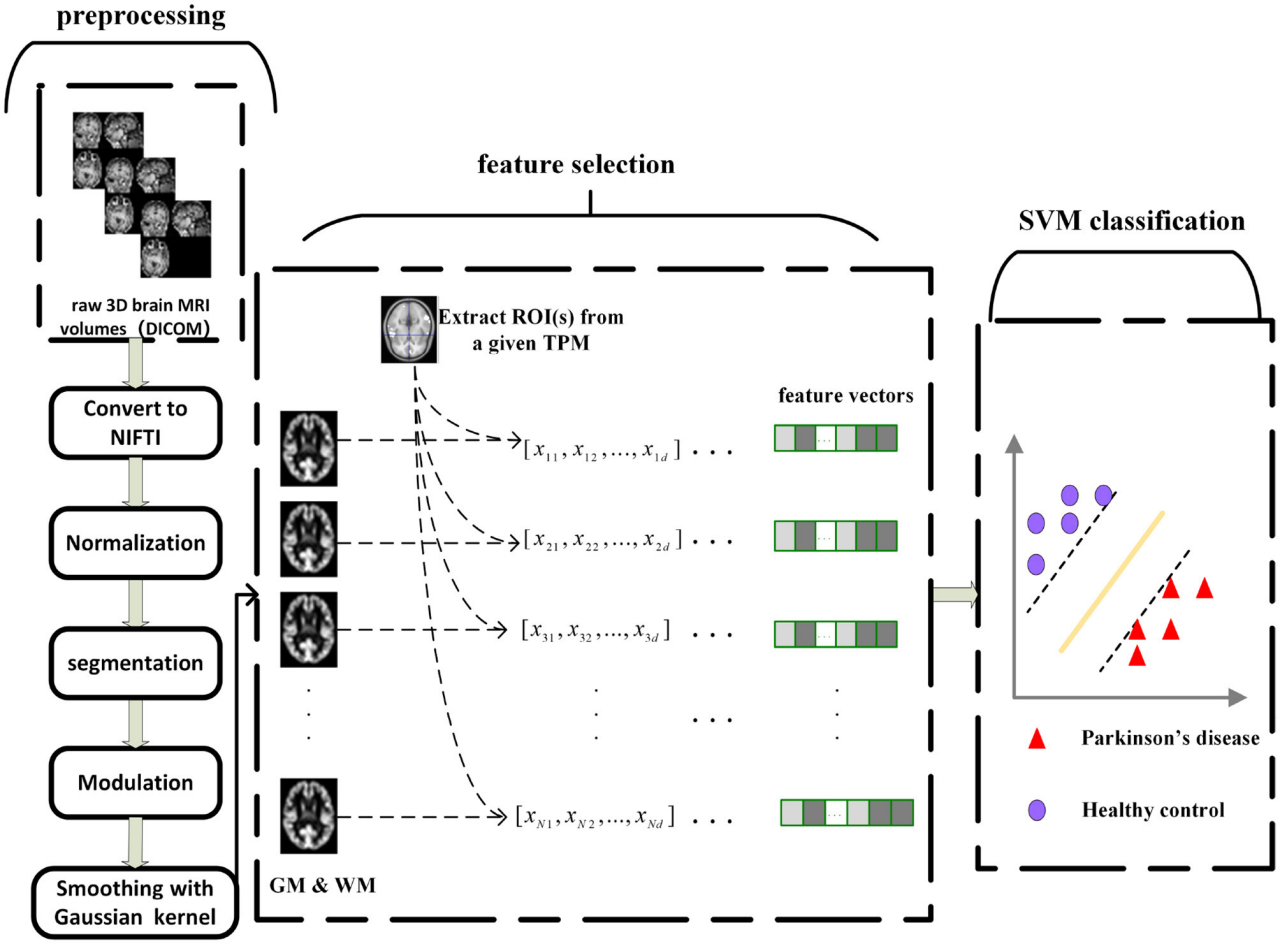
The processing pipeline for machine learning in our study.

The whole classification framework, including data preprocessing, feature selection, and classification is presented in Figure 1. As shown in the figure, the whole experiment is divided into three modules: data preprocessing, feature selection, and feature classification. Among these modules, the purpose of data preprocessing is to eliminate the effects of geometric distortion, intensity imbalance, and noise in MRI as a result of human factors or defects in the acquisition equipment itself. Since most studies based on neuroimaging have limited data with very high dimensionality, the classification on original data directly without feature selection will lead to high computational cost and over-fitting problem, which make that machine learning models have poor generalization ability. In order to reduce the risk of overfitting of the classification model and enhance the interpretability of the machine learning model, it is necessary to reduce the dimension of the data and evaluate the importance of features with a feature selection technique. Finally, a linear SVM was used to construct the classification model, which showed significant performance for small data-sets (Vapnik, 1999; Anguita et al., 2011; Peng et al., 2015).

### 3.1. MRI Data Preprocessing

Voxel-based morphometry (VBM) is an advanced and powerful quantitative MRI procedure designed to detect the brain morphological/volumetric changes in brain diseases. Study has shown (Farokhian et al., 2017) that a VBM analysis using the Computational Anatomy Toolbox (CAT12) is more robust and accurate in terms of volumetric alterations than using older versions of VBM8 toolbox. Since the purpose of this study is to conduct a horizontal comparison with previous studies based on VBM8, we still considered to adopt VBM8 to exclude the impact of irrelevant factors and keep consistency with previous founding.

The original Digital Imaging and Communications in Medicine (DICOM) images were converted to 3D NIFTI format using MRIcron (https://people.cas.sc.edu/rorden/mricron/index.html). Further image processing was performed based on the voxel-based morphometry (VBM) technique with the VBM8 toolbox (http://dbm.neuro.uni-jena.de) in conjunction with Statistical Parametric Mapping software (SPM8, Wellcome Institute of Neurology, University College London, UK) (http://www.fil.ion.ucl.ac.uk/spm/) running on a Matlab R2013b platform. The preprocessing included the following steps: (1) Spatial normalization was performed by resampling and reorienting 3D T1-weighted MR volume of each subject using T1 templates from SPM8. (2) Gray matter (GM), white matter (WM), and cerebrospinal fluid (CSF) were obtained by segmentation of all normalized volumes. Meanwhile, in order to maintain local tissue volume, modulation was performed simultaneously in the segmentation process. (3) Spatial smoothing was performed with a 6 mm isotropic full width at half maximum Gaussian kernel.

### 3.2. Statistical Analysis

In this study, we applied a two-sample *t*-test to extract group-level brain changes from GM and WM separately. Whole brain analysis was performed with a threshold of uncorrected *p* < 0.05 and cluster size ≥ 100, which have been verified to effectively balance false positives and negatives (Altamura et al., 2017; Lojowska et al., 2018; Mishra et al., 2019). Finally, the statistical map was utilized as a 3D mask template to extract different voxels from the corresponding smoothed GM and WM.

### 3.3. Feature Selection and Classification

As one of the most important techniques of machine learning, feature selection has been used to analyze neuroimaging because it enables the user to conveniently identify areas that differ among different groups. In this study, we chose four different feature selection methods to construct the learning framework, and we explored their classification performance. The detailed algorithm design is given in the Supplementary Material. The four feature selection methods, namely, stability selection (Meinshausen and Bühlmann, 2010), ReliefF (Kira and Rendell, 1992; Robnik-Šikonja and Kononenko, 2003), spectral feature selection (Zhao and Liu, 2007), and recursive feature elimination with linear SVM (Guyon, 2001), are widely used in the field of neuroscience (Mwangi et al., 2013; Tohka et al., 2016). Subsequently, SVM, as a powerful classifier based on the principle of structural risk minimization (Sain, 1997), is used to construct classification models. The main principle of SVM is to find a hyperplane that maximizes the margin between two types of objects to separate measurements.

### 3.4. Performance Evaluation

We utilized 5-fold cross-validation to verify the effectiveness of the classification model. We divided the data into five subsets and sequentially designated each one as a test set to test the performance of the model trained on the remaining data. In addition, to further prove the stability of the experimental results, we conducted 10 iterations of each cross-validation. Finally, the performance of the learning model was evaluated by calculating the sensitivity, specificity, and accuracy, which were defined as:

$$Sensitivity=\frac{TP}{TP+FN}$$

$$Specificity=\frac{TN}{TN+FP}$$

$$Accuracy=\frac{TP+TN}{TP+FP+FP+FN}$$

Suppose *N* represents the number of patients, and *M* refers to the number of healthy individuals. *N*′ is the number of patients correctly classified, and *M*′ is the number of healthy controls correctly classified. *TP, TN, FP*, and *FN* are represented as follows:

$$TP=\frac{N^{\prime }}{N}$$

$$TN=\frac{N-N^{\prime }}{N}$$

$$FP=\frac{M^{\prime }}{M}$$

$$FN=\frac{M-M^{\prime }}{M}$$

Accuracy, calculated as an arithmetic mean of sensitivity and specificity, was used to measure the overall performance of the model in PD classification. In general, sensitivity and specificity affect each other, and an increase in one of them will inevitably lead to a decrease in the other.

## 4. Experimental Results and Discussion

In this section, we evaluated and compared the classification performance of four different feature selection methods for GM, WM, and GM+WM to investigate the stability of machine learning approaches. For GM or WM, the input data are GM or WM individually, and for GM+WM, the GM and WM have been stacked together and input to training machine learning models. Furthermore, distinguishing brain regions have been identified by feature selection techniques and compared with the findings of conventional statistical analysis.

All machine learning experiments were conducted on Windows with a 3.1GHz Intel Core i5 processor (4 cores) and 12GB RAM. The Python programming language was utilized for the experiments. In this study, spectral feature selection (SPEC) and ReliefF methods were implemented through *scikit-feature* (Li et al., 2018). Stability selection (STABLASSO) was implemented in *stability-selection* (Meinshausen and Bühlmann, 2010). Recursive feature elimination (RFE) was implemented in *scikit-learn* (Pedregosa et al., 2012). The relevant method parameters were set as follows: (1) ReliefF: The number of neighbors samples *k* is set to 5. (2) STABLASSO: The LASSO *estimator* was calculated. We used *m* = 1,000 iterations. The experiments were performed with a threshold value of π_*th*_ = 0.085. (3) SVM-RFE: Linear SVM is used as the *estimator*, and the *step* parameter is set to 0.1. Leave the rest of the parameters as default values. Notably, the parameter *C* of the linear-SVM classifier played an important role in all experiments. In this study, we tested different values of this parameter, with *C* = {10^−3^, 10^−2^, 10^−1^, 10^0^, 10^1^, 10^2^, 10^3^}, and we found that the performance of linear SVM was best when *C* = 1 for both GM and WM. Since each feature selection method requires to assign the number of features (for SPEC, ReliefF, and RFE) or the minimum threshold of stability scores (for STABLASSO) to determine the number of retained features, we retained different numbers of features based on the percentage of voxels in an iterative manner and ultimately selected the number of features with the best classification performance as the optimal number of features for SPEC, ReliefF, and RFE. For STABLASSO, due to its expensive computational cost, the fixed threshold (threshold = 0.085) of stability scores has been used to determine the optimal number of features in this study. The optimal numbers of features (retained features) selected under different methods are listed in Tables 2–**4**.

**Table 2:** The classification performance (in percentage) of four machine learning methods on GM.

			**GM**			
	**RetainedFeas**	**OptimalFeas**	**Sen**	**Spec**	**Acc**	**AUC**
SPEC	9,396	8,045	86.93 ± 2.41	91.36 ± 2.94	88.61 ± 1.86	89.15 ± 2.53
ReliefF	9,396	7,809	87.29 ± 3.54	**92.24± 3.69**	**89.58 ± 2.39**	**89.77 ± 3.58**
RFE	5,285	2,841	**92.42 ± 2.62**	85.85 ± 2.46	88.56 ± 1.99	89.14 ± 2.55
STABLASSO	3,547	2368	90.41 ± 3.21	79.55 ± 2.39	84.98 ± 1.99	84.98 ± 2.98

* *RetainedFeas, The number of features retained by different feature selection methods; OptimalFeas, the optimal feature subsets selected from retained features; Sen, sensitivity; Spec, specificity; Acc, accuracy; AUC, The area under ROC curve. The best performance for each indicator is shown in bold*.

### 4.1. Stability Analysis Based on Classification Performance

Tables 2–4 have listed the classification performance of the four feature selection methods on GM, WM, and GM+WM. As shown in Tables 2, 3, the highest classification performance was 92.24% (specificity), 92.42% (sensitivity), 89.58% (accuracy), and 89.77% (AUC) for GM and 71.93% (specificity), 74.87% (sensitivity), 71.18% (accuracy), and 71.82% (AUC) for WM. Therefore, machine learning methods on GM have achieved better classification performance than those on WM, which has indicated that PD has a greater effect on the brain regions of GM than WM (Feldmann et al., 2010; Ji et al., 2010; Kang et al., 2015). As seen in Table 4, ReliefF method achieved the best classification performance: 89.66% (sensitivity), 80.01% (specificity), 84.92% (accuracy), and 84.84% (AUC). In addition, it can be seen that GM+WM was classified more effectively than WM but less effectively than GM. In addition, the variation curves of accuracy with the number of features under the SPEC, ReliefF, and RFE methods are provided in Supplementary Figures S1, S2.

**Table 3:** The classification performance (in percentage) of four machine learning methods on WM.

			**WM**			
	**RetainedFeas**	**OptimalFeas**	**Sen**	**Spec**	**Acc**	**AUC**
SPEC	9,922	6,048	72.18 ± 3.77	69.35 ± 3.54	70.39 ± 2.33	70.77 ± 3.65
ReliefF	9,922	5,457	**74.87 ± 3.45**	68.77 ± 2.95	**71.18 ± 2.09**	**71.82 ± 3.28**
RFE	7,937	3,360	69.85 ± 4.24	66.31 ± 4.08	68.16 ± 3.49	68.08 ± 4.16
STABLASSO	5,073	3,233	69.30 ± 4.28	**71.93 ± 2.25**	70.11 ± 2.90	70.62 ± 3.87

*The best performance for each indicator is shown in bold*.

**Table 4:** The classification performance (in percentage) of four machine learning methods with GM and WM.

			**GM + WM**			
	**RetainedFeas**	**OptimalFeas**	**Sen**	**Spec**	**Acc**	**AUC**
SPEC	10,740	4,115	89.20 ± 2.31	79.26 ± 4.66	84.20 ± 3.01	84.23 ± 3.74
ReliefF	10,424	3,996	**89.66 ± 3.84**	80.01 ± 3.10	**84.92 ± 1.80**	**84.84 ± 3.48**
RFE	11,372	5,647	86.29 ± 3.07	78.47 ± 4.30	82.37 ± 3.26	82.38 ± 3.96
STABLASSO	4,425	2,753	87.43 ± 2.50	**80.67 ± 1.72**	84.25 ± 1.43	84.05 ± 1.88

*The best performance for each indicator is shown in bold*.

**Figure 2:**
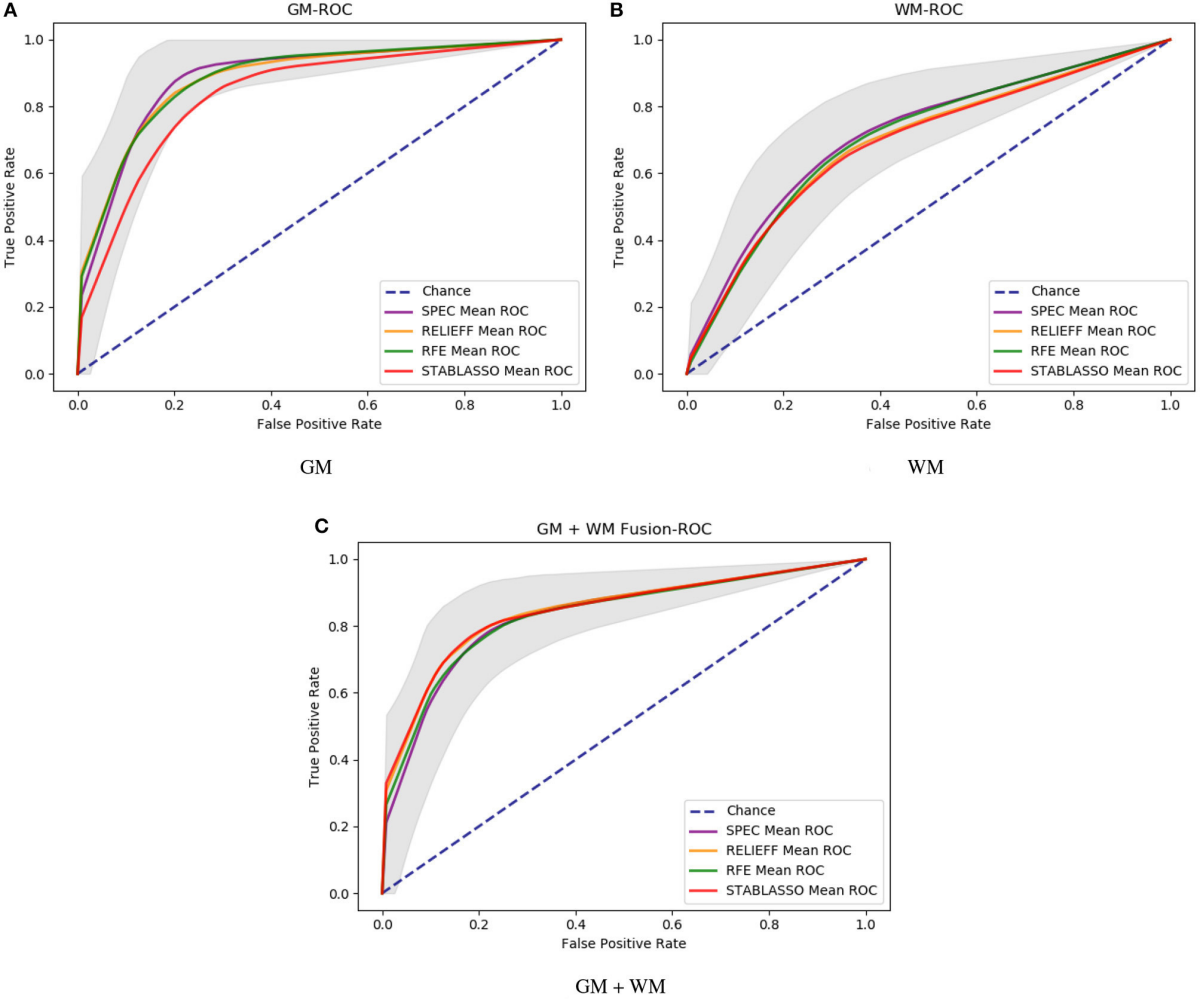
The ROC curve of different methods with different modalities. **(A)** ROC curves for GM; **(B)** ROC curves for WM; **(C)** ROC curves for GM+WM.

It should be further noted in Table 2 that the classification accuracy for GM had a maximum of 89.58% and a minimum of 84.98%, with a difference of approximately 4.6%. For WM, the highest accuracy was 71.18%, and the lowest accuracy was 68.16%, for a small difference of approximately 3%. For GM+WM classification experiment, the highest classification accuracy was 84.92%, and the lowest was 82.37%, for a difference of approximately 2.5%. These small differences have demonstrated that patients with PD can be stably distinguished from HCs with machine learning techniques. Furthermore, the receiver operating characteristic (ROC) curves have been illustrated in Figure 2. Generally, the effect of a classifier can also be evaluated by the area under the ROC curve (AUC). The machine learning methods whose AUCs are closest to 1 can be considered to have the best performance. The AUCs are given in Tables 2–4, respectively. Similar to the accuracy, the AUCs of different methods on different modalities also have subtle differences, and the ROC curves of different methods in GM/WM/GM+WM were approxiimately the same.

### 4.2. Stability Analysis Based on Identified Brain Regions

In this section, we intended to explore the optimal feature subsets from retained features for each feature selection method, which are expected to be PD-associated brain changes in GM/WM. For this purpose, the most distinguished brain regions were defined as the optimal feature subsets if their frequency being selected in cross validation is greater than 80%. For machine learning, the original dimensions of input data for GM and WM are 11,745 and 19,844, respectively. The number of retained features for feature selection and optimal feature subsets from retained features have been listed in Tables 2–4. Moreover, we have compared the distinguished brain regions of GM and WM based on the optimal feature subsets with the findings of previous studies and traditional statistical analysis.

#### 4.2.1. Gray Matter Analysis

There have been numerous prior studies using gray matter to analyze Parkinson's disease. Long et al. (2012) found that the volume of GM was significantly reduced in the Paracentral lobule (PCL) and significantly increased in the Precentral gyrus (PreCG) and the bilateral Posterior cingulate gyrus (PCG) in the PD group. Adeli et al. (2016) found that GM tissue changes play the most important role in PD classification in most regions such as the PreCG, Supplementary motor area (SMA), Superior frontal gyrus (medial, SFGmed), Insula (INS), Median cingulate and paracingulate gyri (DCG), Calcarine fissure and surrounding cortex (CAL), Lingual gyrus (LING), Fusiform gyrus (FFG), Postcentral gyrus (PoCG), Superior parietal gyrus (SPG), Caudate nucleus (CAU), Lenticular nucleus, putamen (PUT), Lenticular nucleus, pallidum (PAL), Thalamus (THA), Superior temporal gyrus (STG), Middle temporal gyrus (MTG), and Inferior temporal gyrus (ITG). Peng et al. (2017) found in their study that the most sensitive GM biomarkers for distinguishing between patients with PD and healthy controls were the SPG, PCL, and Parahippocampal gyrus (PHG). Santos et al. (2013) carried out VBM on GM and observed atrophy in the following brain regions: OLF, Middle frontal gyrus (orbital part, ORBmid), Inferior frontal gyrus (orbital part, ORBinf), Superior frontal gyrus, (medial orbital, ORBsupmed), MTG, STG, INS, and PreCG. On the other hand, Xia et al. (2013) identified the STG, Superior occipital gyrus (SOG), SPG, Middle frontal gyrus (MFG), INS, PHG, and Amygdala (AMYG) as regions with altered GM volume using VBM. Jia et al. (2015) found in their study that GM volume decreased in the Superior frontal gyrus (orbital part, ORBsup), MFG, MTG, ITG, SPG, Inferior parietal, but supramarginal and angular gyri (IPL), Angular gyrus (ANG), and CAU, while GM volume increased in the ORBinf, MOG, Anterior cingulate and paracingulate gyri (ACG), PAL, PUT, and Hippocampus (HIP). Liu et al. (2018) found that the GM/WM brain regions contributing most to PD classification were mainly concentrated in the MOG, PUT, CAU, ORBsup, ACG, PreCG, HIP, Precuneus (PCUN), and PoCG. Rana et al. (2017) further improved the performance of PD classification by defining a fusion feature descriptor (FFD) to capture information and interrelationships between GM and WM tissues simultaneously. Ultimately, they found that the most discriminative brain regions were mainly located in the HIP, DCG, Inferior frontal gyrus (triangular part, IFGtriang), PreCG, MFG, ORBmid, and ACG. These distinguished brain regions have been listed in Supplementary Table S2.

In this study, the abnormal brain regions have been illustrated in Figure 4 and reported based on AAL (Automated Anatomical Labeling atlas) with 116 brain regions (Tzourio-Mazoyer et al., 2002). We found that the SPEC method identified the largest number of features based on brain GM analysis, followed by ReliefF and RFE, and STABLASSO found the fewest, as seen in Supplementary Table S5. Furthermore, we note that the significant brain regions found by SPEC included all the brain regions found by the other three methods. The brain regions found commonly by all four methods included the PCUN, CAU, STG, MFG, MOG, MTG, INS, SFGdor, ORBinf, IPL, and ANG. Except SFGdor, other brain regions have been reported in the previous studies. Furthermore, the findings of statistical analysis on GM have been listed in Supplementary Table S3. It is easy to find that most brain regions found by statistical analysis also have been identified by machine learning methods, except CAL with the cluster size 113. The possible reason is that the cluster size is too small or the changes in this brain region is subtle.

**Figure 3:**
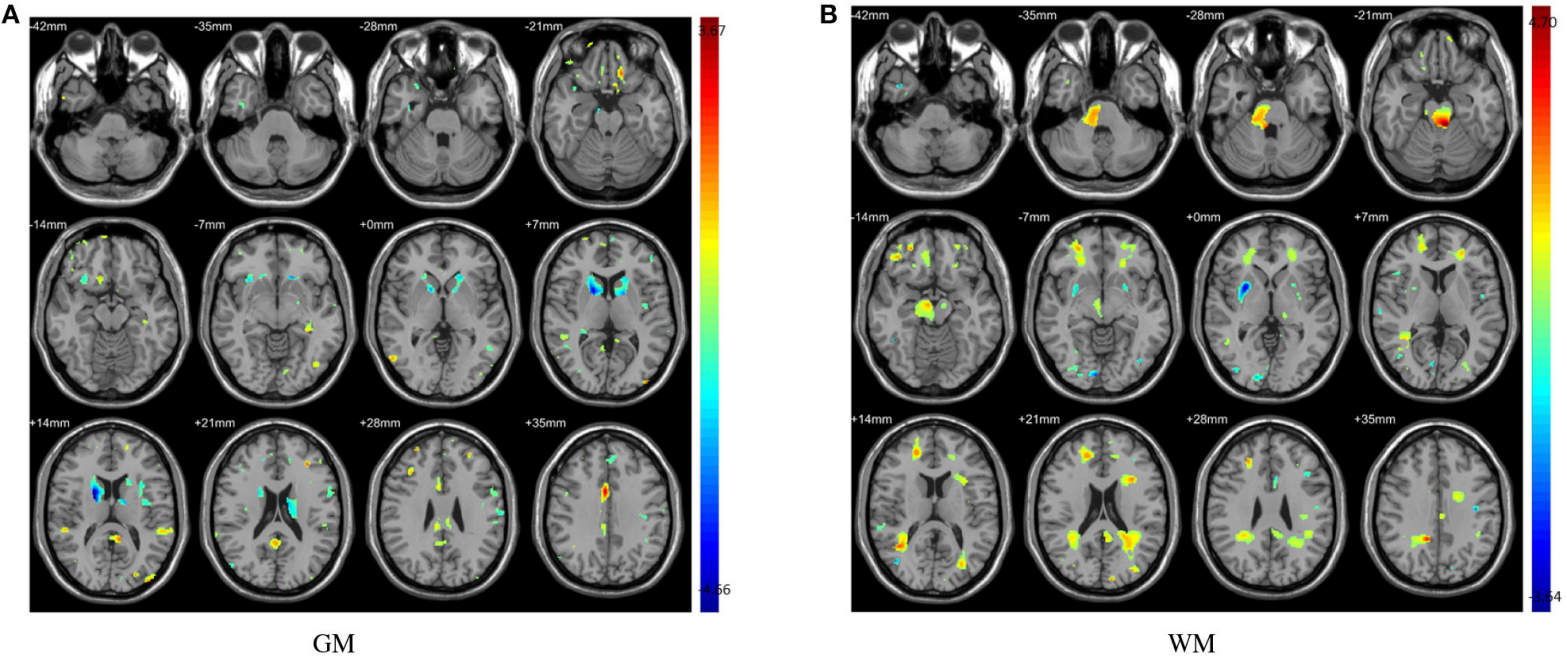
The abnormal brain regions identified by two sample *t*-test. Color bars indicate *t*-value. **(A)** Abnormal regions of GM; **(B)** Abnormal regions of WM.

**Figure 4:**
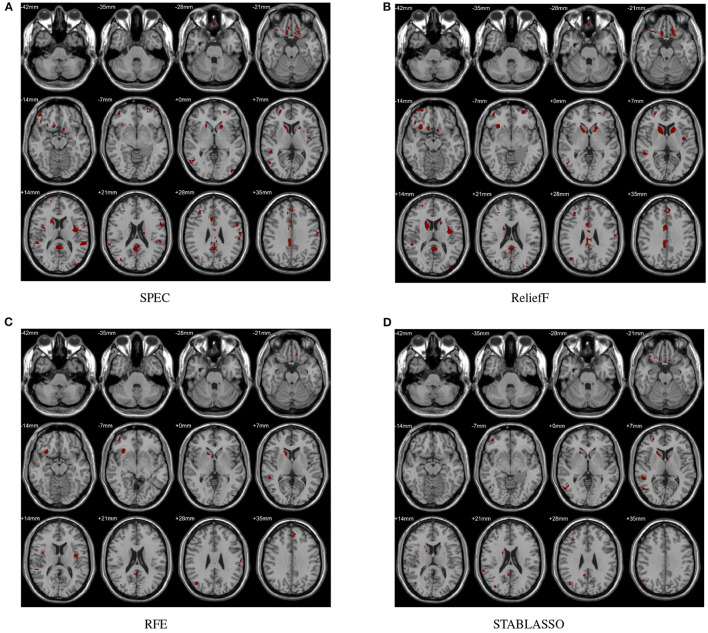
Biomarkers identified on GM by different methods. **(A)** Biomarkers identified by SPEC; **(B)** Biomarkers identified by ReliefF; **(C)** Biomarkers identified by RFE; **(D)** Biomarkers identified by STABLASSO.

#### 4.2.2. White Matter Analysis

Similarly, a great number of studies have been conducted to analyze the potential value of WM in the classification of PD. Ding et al. (2011) found that the changes in WM tissue volume in most regions, including SOG, Inferior occipital gyrus (IOG), PCG, and PCL, contributed to the improvement of overall classification performance. Peng et al. (2017) also found that the most sensitive WM biomarkers for distinguishing between patients with PD and healthy controls were the ITG, FFG and MFG. Long et al. (2012) found that brain regions showing WM volume changes were mainly located in the PreCG, ORBinf, Rolandic operculum (ROL), OLF, HIP, AMYG, PoCG, and CAU. On the other hand, the MOG, IPL, PAL, THA, ITG, CAU, and PUT were identified by Adeli et al. (2016) as regions whose WM volume was affected. Rana et al. (2017) further improved the performance of PD classification by defining a fusion feature descriptor (FFD) to capture information and interrelationships between GM and WM tissues simultaneously. Ultimately, they found that the most discriminative brain regions were mainly concentrated in the HIP, DCG, IFGtriang, PreCG, MFG, ORBmid, and ACG. Liu et al. (2018) found that the GM/WM brain regions most contributing to PD classification were mainly concentrated in the MOG, PUT, CAU, ORBsup, ACG, PreCG, HIP, PCUN, and PoCG. These distinguished brain regions have been listed in Supplementary Table S2.

**Figure 5:**
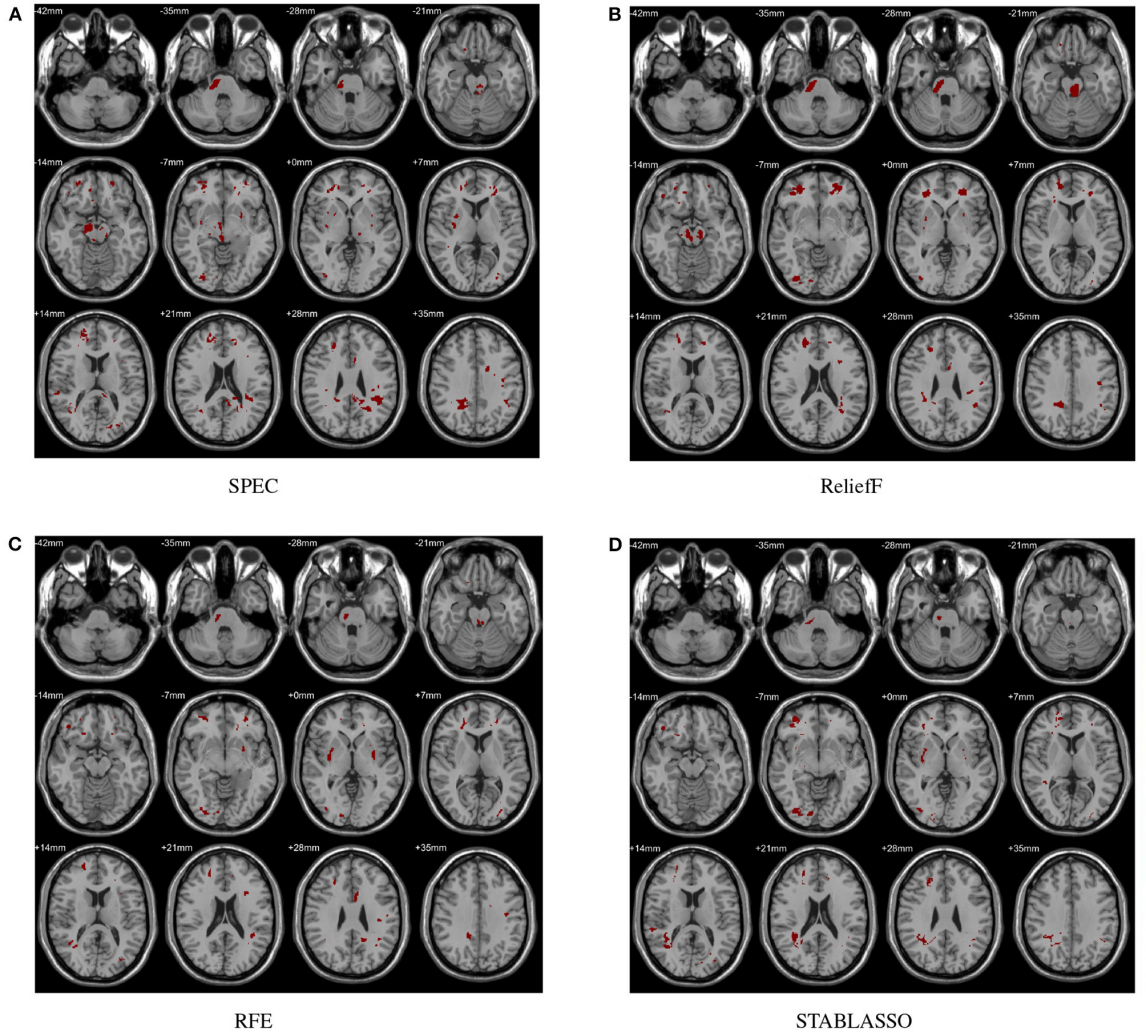
Biomarkers identified on WM by different methods. **(A)** Biomarkers identified by SPEC; **(B)** Biomarkers identified by ReliefF; **(C)** Biomarkers identified by RFE; **(D)** Biomarkers identified by STABLASSO.

The distinguished brain regions on WM identified by machine learning have been shown in Figure 5 and reported based on AAL listed in Supplementary Table S6. The common regions found by four feature selection include PoCG, ORBmid, SFGdor, IOG, and LING. Except for SFGdor and LING, other regions have been reported in previous findings. The results detected by statistical analysis have been listed in Supplementary Table S4 and shown in Figure 3. Similarly, most regions also have been found by machine learning methods, while some small regions have not been found, such as INS (cluster size = 99), IPL (cluster size = 109), ANG (cluster size = 47), IFGoperc (cluster size = 39), and SPG (cluster size = 57).

### 4.3. Discussion

Parkinson's disease (PD) is the most common neurodegenerative disorder (Kalia and Lang, 2015). At its core, it is a movement disease with early prominent death of dopaminergic neurons in the substantia nigra pars compacta (SNpc) (Kalia and Lang, 2015). Pathologically, it is also characterized by accumulation of misfolded –synuclein, which is found in intra-cyto-plasmic inclusions called Lewy bodies (Balestrino and Schapira, 2020). The gold standard for diagnosis of PD has been the presence of SNpc degeneration and Lewy pathology at post-mortem pathological examination (Kalia and Lang, 2015). It has been reported that the damage of substantia nigra correlated with basal ganglia dysfunction for patients with PD (Vitali et al., 2020). In our study, the changes of the caudate nucleus (CAU) in basal ganglia have also been found in GM, which have emphasized the relationship between substantia nigra and basal ganglia again. However, the relationship between the nigral anatomical changes, evaluated as structural alterations or neuromelanin signal decrease and the dopaminergic nigro-striatal function needs to be further clarified (Prange et al., 2019).

Recently, the detection and diagnosis of neurodegenerative diseases have attracted a great deal of research interest. There have been a large number of studies on GM and WM changes in patients with PD. They also identified several brain regions significantly affected by PD. Zheng et al. (2019) found that brain GM changes in the left anterior insula were associated with mild cognitive impairment in PD. In González Redondo et al. (2014), bilateral areas of atrophy in the middle frontal gyrus and bilateral GM loss in the medial-superior frontal gyrus were reported in patients with PD. Significantly increased WM density in the occipital lobes, posterior cingulate gyrus, and paracentral lobule was found in PD with olfactory dysfunction compared with healthy controls (Ding et al., 2011). As reported in Niethammer et al. (2013), a significant relationship between the loss of dopaminergic input to the caudate nucleus and the expression of a cognition-related disease network was found in unmedicated patients with PD. Gao et al. (2017) revealed that subcortical atrophy (for example, in the limbic lobe) was associated with impaired memory in patients with PD.

Although machine learning techniques have attracted great interest in the field of neuroscience, some researchers still remain skeptical about the stability and interpretability of machine learning outputs. As a result, instead of developing a new machine learning framework, this study focuses on horizontal comparison of different machine learning methods and vertical comparison between machine learning techniques and statistical analysis to enhance the credibility of machine learning. For this purpose, we applied a typical machine learning framework with feature selection and feature classification to distinguish PD from HCs. Four popular feature selection methods were utilized to find a set of relevant and nonredundant features. A support vector machine was employed to build the classification models. An sMRI dataset was constructed to evaluate the performance of machine learning methods. Experimental results showed that the maximum accuracy levels based on GM and WM were 89.58 and 71.18%, respectively, which highlight the role of feature information in improving classification accuracy. Machine learning techniques are more effective than traditional statistical methods at extracting meaningful information from high-dimensional structural images and achieving stable classification results. Moreover, machine learning methods overcome the shortcomings of traditional statistical methods and extend simple analysis of differences from the group level to the individual diagnostic level. In addition, machine learning techniques identified many GM and WM brain changes that could effectively distinguish patients with PD from controls. Through further comparison, most brain changes identified by machine learning techniques were found to be consistent with previous studies of structural MRI. At the same time, we also found some brain regions have not been reported in previous studies on structural MRI, such as SFGdor for GM and LING, SFGdor for WM, while SFGdor and LING have been confirmed in other modality studies. Li et al. (2020) has pointed out the reduced betweenness centrality in right SFGdor for PD. Auning et al. (2014) has recovered higher radical diffusivity in LING on DTI when compared PD and NC. Our findings support that SFGdor and LING may be the important biomarkers in PD.

In light of the above analysis, the major contributions to the field by this study concern the following issues: (i) Stability—The horizontal comparison of different machine learning methods has demonstrated that machine learning techniques can robustly classify PD and HCs; (ii) Interpretability—In terms of feature selection, machine learning techniques are helpful in identifying the distinct brain changes that can be used as potential biomarkers of PD; (iii) Consistency—Based on the vertical comparison, the brain changes found by machine learning techniques are partly consistent with those found by statistical analysis. Despite superior performance and ease of utilization, the machine learning method used in the paper also has a few disadvantages: (i) Although this study investigated classification performance on GM and WM by simply concatenating the GM and WM data, the correlation between the two types of tissues was ignored; such information could be helpful in improving the performance of machine learning. (ii) The dataset used in this study is limited and needs to be further expanded to make the results more generalizable. (iii) Machine learning techniques were used to classify PD solely on the basis of sMRI, and its needs; the same techniques need to be applied to more neuroimaging modalities to prove their effectiveness. (iv) Machine learning techniques need to be extended to other disease investigations to prove their universality. (v) To assess the stability of the discriminative brain regions, we have focused only on the brain regions that were identified by all four machine learning methods and reported by previous research work at the same time. However, the biomarkers of PD may also include some brain regions identified by our machine learning methods but not reported by previous findings based on conventional statistical analysis.

In future research work, this study should be expanded upon in the following directions: (i) It cannot be denied that the pre-processing of MRI data is crucial for the subsequent data analysis and VBM analysis with different softwares and versions are controversial until now. As pointed out in Farokhian et al. (2017), CAT12 has shown great advantage to detect the volumetric alterations compared with VBM8, so it necessitates to conduct further investigation based on CAT12 in the future. (ii) In order to fully consider the correlation of different modalities, it is necessary to integrate multi-modal features to capture the information and interrelationship simultaneously. (iii) In order to further demonstrate the universality of machine learning, it might be worthwhile to study the consistency of the results of machine learning techniques on a larger dataset.

## 5. Conclusion

This study was designed to analyze the changes in GM and WM brain tissue in PD. In this study, we adopted four popular feature selection methods to extract a group of the most relevant and nonredundant features, and we then used linear support vector machines to construct classification models for WM and GM. In the experiment, we have achieved stable and satisfactory classification performance on both GM and WM. The brain changes identified by feature selection partially corresponded to previous findings, such as PCUN, CAU, STG, MFG, MOG, MTG, INS, ORBinf, IPL, ANG in GM and PoCG, ORBmid, and IOG in WM, which proved the stable performance of machine learning. Furthermore, machine learning also found commonly the brain changes in SFGdor and LING, which have been proved in other studies. These findings have not only demonstrated the good stability of machine learning technique, but also the superior ability to explore the brain changes, and these brain changes can be regarded as potential biomarkers of PD. These potential biomarkers can help clarify the neurological mechanisms of PD. Moreover, machine learning techniques can provide a decision system for the diagnosis of PD and can be generalized to the diagnosis of other neurodegenerative diseases.

## Data Availability Statement

Publicly available datasets were analyzed in this study. This data can be found here: Parkinson's Progression Markers Initiative (PPMI), https://www.ppmi-info.org/.

## Ethics Statement

The studies involving human participants were reviewed and approved by the Michael J. Fox Foundation for Parkinson's Research. The patients/participants provided their written informed consent to participate in this study.

## Author Contributions

CS, WZ, HJ and XL have contributed equally to this study. CS, WZ, CL and YT conceived and designed this study, HJ, XL and JK participated in the analysis of MRI dataset, YD, XDY, XY, JZ helped to draft the manuscript. All authors read and approved the final manuscript.

## Funding

This work was supported by Sichuan Science and Technology Program (Grant Nos. 2018JY0272 and 21GJHZ0012), the China Postdoc-toral Science Foundation (Grant No. 2016M592656), the Science and Technology Bureau of ChengDu (Grant No. 2020-YF09-00005-SN) and Erasmus+ SHYFTE Project (Grant No. 598649-EPP-1-2018-1-FR-EPPKA2-CBHE-JP).

## Conflict of Interest

The authors declare that the research was conducted in the absence of any commercial or financial relationships that could be construed as a potential conflict of interest.

## Publisher's Note

All claims expressed in this article are solely those of the authors and do not necessarily represent those of their affiliated organizations, or those of the publisher, the editors and the reviewers. Any product that may be evaluated in this article, or claim that may be made by its manufacturer, is not guaranteed or endorsed by the publisher.
