# Supplementary material for: Stability Evaluation of Brain Changes in Parkinson's Disease Based on Machine Learning
Source: Front Comput Neurosci. 2021 Oct 26;15:735991. doi: 10.3389/fncom.2021.735991 (PMC8594429; doi:10.3389/fncom.2021.735991)
Supplement: Supplementary file 1 [file Data_Sheet_1.PDF]

Supplementary Material

1 SUPPLEMENTARY FEATURE SELECTION ALGORITHM

1.1 Stability Selection

Stability selection is a new method that combines subsampling with feature selection algorithms. The key idea of this method is to subsample training data randomly many times based on feature selection with different values of the regularization parameters and select the features that are most frequently selected in different subsamples. The stability selection process is as follows:

Stability Selection algorithm:

Input: a set of regularization parameters Λ, the number of iterations m, and the cutoff threshold value π_{th}.

1. For each regularization parameter $\lambda \in \Lambda$:

– Draw a subsample of training data X_i of size $\lfloor \frac{N}{2} \rfloor$, where N is the number of training data, without replacement.

– Run the Lasso on X_i using parameter λ and obtain β^i, where β is the training sparse parameter for given classifier. Keep the selected features $S^{\lambda}(X_i) = \{f : \beta_f^{\lambda} \neq 0\}$.

2. Repeat step (1) m times. Then calculate the probability of each feature $f_i, i = 1, ..., p$ being selected in random resampling based on the following equation.

$$\prod_k^{\lambda} = \frac{1}{M} \sum_{i=1}^{M} 1 \left\{ k \in S^{\lambda}(X_i) \right\}$$

where the $1\left\{.\right\}$ is the indicator function.

3. For each feature f_i, the corresponding stability score is calculated and only features with scores greater than π_{th} are retained.

$$S_{stable} = \left\{ f : \max_{\lambda \in \Lambda} (\prod_f^{\lambda}) \geq \pi_{th} \right\}$$

Output: Set S_{stable}.

1.2 Relief Family Of Algorithms

Relief, a popular algorithm for feature quality estimation, does not need to assume the conditional independence of features as most heuristic algorithms do and is therefore appropriate for problems that may involve much feature interaction. The quality of each feature is estimated according to the degree to which the eigenvalues differ between instances that are near each other. In this study, we used an extension of this algorithm, known as ReliefF; ReliefF is a more robust improvement on Relief.

The ReliefF algorithm randomly selects a sample X_i from the training sample set each time but then determines the k nearest neighbor samples of X_i from the sample set of the same class as X_i, called nearest

hits H_j, and the k nearest neighbor samples from each sample set belonging to different classes than X_i, called nearest misses $M_j(C)$. Then, the weight of each feature is updated according to the following formula:

$$W(F) = W(F) - \sum_{j=1}^{k} diff(F, X_i, H_j)/(m.k) +$$

$$\sum_{C \neq class(X_i)} [\frac{P(C)}{1 - P(class(X_i))} \sum_{j=1}^{k} diff(F, X_i, M_j(C))]/(m.k)$$

where $diff(F, X_1, X_2)$ is the difference between X_1 and X_2 on feature F. $M_j(C)$ represents the jth nearest neighbor sample in class $C \neq class(X_i)$.

$$diff(F, X_1, X_2) = \begin{cases} \frac{|X_1[F] - X_2[F]|}{\max(F) - \min(F)} & \text{if F is continuous.} \\ 0 & \text{if F is discrete and } X_1[F] = X_2[F]. \\ 1 & \text{if F is discrete and } X_1[F] \neq X_2[F]. \end{cases}$$

Finally, all features are sorted by weight value.

1.3 Spectral Feature Selection

Inspired by the concept of graphs, Zhao and Liu proposed an unsupervised feature selection method. Unsupervised methods depend only on the inherent structure of the data and not on class labels. Thus, the spectral feature selection method can reveal the structure of data geometrically.

The core idea of the spectral feature selection method is to construct an undirected weighted graph G. Here, the sample similarity matrix W that expresses the weight matrix is calculated. Then, the corresponding degree matrix (D), Laplace matrix (L) and normalized Laplace matrix $(\pounds)$ are derived from W (**?**). Finally, the scoring function $(\varphi(f_i))$ for each feature vector (f_i) is calculated as follows:

$$\varphi(f_i) = \frac{\hat{f_i^T} \pounds \hat{f_i}}{1 - (\hat{f_i^T} \xi_0)^2}$$

where $\hat{f_i} = \frac{D^{-1/2} f_i}{\|D^{-1/2} f_i\|}$ is a normalized weighted feature vector and λ_i and $\xi_i (0 \leq i \leq N-1)$, respectively, correspond to the eigenvalues and eigenvectors of the spectral decomposition of $(\pounds)$. The importance of each feature f_i is measured by the value of $\varphi(f_i)$. A smaller value of $\varphi(f_i)$ means that feature f_i has higher smoothness over graph G. Features with high smoothness will assign similar values to samples from G that are close to each other. Such features are regarded as important.

1.4 Support Vector Machine Recursive Feature Elimination

The support vector machine recursive feature elimination (SVM-RFE) method was first proposed by Guyon et al. In the present study, we selected nested feature subsets by sequential backward elimination. Then, the selected feature subset was used to train the model, and the feature ranking scores was calculated according to the coefficients of the weight vector W of linear SVM. Finally, the corresponding features with a ratio of 0.1 were removed. The above procedure was repeated recursively until the number of remaining

features reached the required number of features. The scoring function was defined as $c_i = (w_i)^2$, where w_i represents the corresponding component of the weight vector W.

$c_i = (w_i)^2$, a ranking criterion, was used to remove the features that have the least impact on the objective function. The objective function took the following form:

$$J = argmin((1/2)\|W\|^2)$$

1.5 Feature Classification

SVM was originally proposed for binary classification, and the hyperplane (decision boundary) was determined by the following equation:

$$W^T x + b = 0 \tag{S1}$$

where $W \in R^k$ is weight vector and $b \in R^k$ is bias. Equation (2) gives the original binary optimization solution problem:

$$\min_{W,b,\xi} \frac{1}{2}||W||^2 + C\sum_{i=1}^{n} \xi_i \tag{S2}$$

s.t.

$$y_i \left\{ W^T x_i + b \right\} \geq 1 - \xi_i \tag{S3}$$

where ξ_i represents slack variables required to tolerate misclassification with $\xi_i \geq 0, i = 1, 2, 3, ..., N$, while $C > 0$ is a tuning parameter used to govern the tradeoff between minimizing the misclassification rate and maximizing the margin width of the training dataset.

2 SUPPLEMENTARY TABLES AND FIGURES

Table S1. Regions of interest (ROIs) included in the AAL-atlas.

Regions	Abbreviations	Regions	Abbreviations
Superior frontal gyrus, dorsolateral	SFGdor	Superior frontal gyrus, orbital part	ORBsup
Middle frontal gyrus	MFG	Middle frontal gyrus, orbital part	ORBmid
Inferior frontal gyrus, opercular part	IFGoperc	Inferior frontal gyrus, triangular part	IFGtriang
Inferior frontal gyrus, orbital part	ORBinf	Rolandic operculum	ROL
Supplementary motor area	SMA	Olfactory cortex	OLF
Superior frontal gyrus, medial	SFGmed	Superior frontal gyrus, medial orbital	ORBsupmed
Gyrus rectus	REC	Insula	INS
Anterior cingulate and paracingulate gyri	ACG	Median cingulate and paracingulate gyri	DCG
Posterior cingulate gyrus	PCG	Hippocampus	HIP
Parahippocampal gyrus	PHG	Amygdala	AMYG
Calcarine fissure and surrounding cortex	CAL	Cuneus	CUN
Lingual gyrus	LING	Superior occipital gyrus	SOG
Middle occipital gyrus	MOG	Inferior occipital gyrus	IOG
Fusiform gyrus	FFG	Postcentral gyrus	PoCG
Superior parietal gyrus	SPG	Inferior parietal, but supramarginal and angular gyri	IPL
Supramarginal gyrus	SMG	Angular gyrus	ANG
Precuneus	PCUN	Paracentral lobule	PCL
Caudate nucleus	CAU	Lenticular nucleus, putamen	PUT
Lenticular nucleus, pallidum	PAL	Thalamus	THA
Heschl gyrus	HES	Superior temporal gyrus	STG
Temporal pole: superior temporal gyrus	TPOsup	Middle temporal gyrus	MTG
Temporal pole: middle temporal gyrus	TPOmid	Inferior temporal gyrus	ITG
Precentral gyrus	PreCG		

Table S2. The brain regions which have been selected as biomarkers in previous research works on PD classification.

Research Work	GM Regions	WM Regions	P-value
(Ding et al. 2011)	—-	SOG IOG PCG PCL	0.05
(Long et al. 2012)	PreCG↑ PCG↑ PCL↓	PreCG ORBinf ROL OLF HIP AMYG PoCG CAU	0.05
(Santos et al. 2013)	OLF↓ ORBmid↓ ORBinf↓ ORBsupmed↓ MTG↓ STG↓ INS↓ PreCG↓	—-	0.05
(Xia et al. 2013)	STG SOG SPG MFG INS PHG AMYG	—-	0.005
(Jia, et al. 2015)	ORBsup↓ MFG↓ MTG↓ ITG↓ SPG↓ IPL↓ ANG↓ CAU↓ ORBinf↑ MOG↑ ACG↑ PAL↑ PUT↑ HIP↑	—-	0.001
(Adeli al. 2016)	PreCG SMA SFGmed INS DCG CAL LING FFG PoCG SPG CAU PUT PAL THA STG MTG ITG	MOG IPL PAL THA ITG CAU PUT	0.05
(Peng al. 2017)	SPG PCL PHG	ITG FFG MFG	0.05
(Rana et al. 2017)	HIP DCG IFGtriang PreCG MFG ORBmid ACG	HIP DCG IFGtriang PreCG MFG ORBmid ACG	0.05
(Liu et al. 2018)	MOG PUT CAU ORBsup ACG PreCG HIP PCUN PoCG	MOG PUT CAU ORBsup ACG PreCG HIP PCUN PoCG	0.05

* ↑ stands for volume increase; ↓ stands for volume reduction.

Table S3: The abnormal grey matter clusters identified by two-sample t-test.

cluster ID	Region of Interest(ROI)	Brodmann	Size of cluster	MNI coordinates(mm)			P-value	T-value
	AAL			X	Y	Z		
PD>HC								
	REC.R/ORBsup.R	11/47	654	15	28.5	-15	0.0029	2.8293
	ORBsup.L/REC.L/OLF.L	11	658	-13.5	31.5	-24	0.0008	3.247
	ORBmid.R/ORBinf.R	11	835	21	61.5	-21	0.0063	2.5474
	ORBmid.L/ORBsup.L	10	514	-19.5	54	-3	0.0132	2.2576
	MTG.R	37	488	55.5	-66	-1.5	0.0036	2.7548
	DCG.R/PCUN.R/PCG.L PCUN.L/PCG.R	31/23/30	2304	9	-31.5	31.5	0.0025	2.8784
	MOG.L	18/19	606	-40.5	-93	9	0.0007	3.3112

STG.R/MTG.R	-	512	52.5	-36	12	0.0022	2.9208
SFGdor.R/SFGmed.R	-	375	16.5	55.5	18	0.0042	2.6992
SFGdor.R	-	159	25.5	54	12	0.0129	2.2686
STG.L	-	441	-55.5	-36	12	0.0042	2.6956
MFG.R	-	213	36	28.5	27	0.0038	2.7281
DCG.R/ACG.R	24	870	4.5	4.5	37.5	0.0002	3.6736
IPL.R	40	155	39	-49.5	51	0.004	2.7177
MFG.L	-	550	-42	10.5	54	0.002	2.9629
IPL.L	40	117	-51	-55.5	43.5	0.0272	1.9491
SMA.R	-	109	4.5	-12	72	0.0078	2.4643
PCL.L	6	234	-10.5	-13.5	72	0.0021	2.9429
PD<HC							
INS.R	47	312	31.5	19.5	-10.5	0.0007	3.3069
CAU.L/PUT.L	-	1025	-13.5	7.5	3	0.0002	3.719
CAU.R	-	1724	19.5	-1.5	13.5	0	4.6631
MOG.R	-	218	30	-76.5	16.5	0.0134	2.2525
INS.L/PreCG.L/ROL.L IFGoperc.L	13	1072	-34.5	13.5	13.5	0.004	2.7165
INS.R	13	228	36	-13.5	22.5	0.0137	2.2424
CAL.R	19	113	9	-85.5	24	0.0098	2.3777
CAU.L	-	549	-12	-7.5	18	0.0001	3.8505
SFGmed.L/SFGmed.R/SMA.R SMA.L	8/6	1211	-7.5	34.5	43.5	0.0022	2.918
PoCG.L	3	358	-60	-16.5	30	0.004	2.7118
ANG.R	-	186	45	-67.5	46.5	0.006	2.567
PCUN.R	-	202	12	-78	46.5	0.0046	2.6647
PreCG.L	6	174	-46.5	-4.5	42	0.0067	2.5239
PCUN.L	7	306	-12	-51	54	0.0015	3.0549
PreCG.L	6	291	-31.5	-18	61.5	0.0025	2.8779

Table S4: The abnormal white matter clusters identified by two-sample t-test.

cluster ID	Region of Interest(ROI) AAL	Brodmann	Size of cluster	MNI coordinates(mm)			P-value	T-value
				X	Y	Z		
PD>HC								
	THA.L	-	3150	-6	-37.5	-21	0	4.7028
	SFGdor.R/ORBmid.R ACG.R/ORBsup.R/ORBinf.R SFGmed.R/MFG.R	11/47/10	3836	25.5	37.5	30	0.0003	3.5704
	REC.R	-	326	3	39	-15	0.0017	3.0046
	ORBmid.L/ORBinf.L ORBsup.L	11	2402	-25.5	43.5	9	0.0008	3.2592
	MTG.R	31	3329	13.5	-48	34.5	0.0001	3.9279
	MOG.L/PCG.L/SMG.L PCUN.L/IPL.L/ANG.L	19	4784	-9	-91.5	24	0.0002	3.7188
	INS.L/IFGoperc.L	-	1924	-31.5	16.5	19.5	0.0004	3.4831
	DCG.L	24	104	-1.5	-22.5	36	0.004	2.7179
	PreCG.R	6	116	49.5	0	45	0.0062	2.5536
	PCUN.R/SPG.R	-	204	13.5	-54	55.5	0.0039	2.7236
PD<HC								
	PUT.R	-	1086	28.5	4.5	-1.5	0.0005	3.408
	CAL.R/LING.R/IOG.R/FFG.R		984	7.5	-88.5	-4.5	0.0002	3.6431
	PUT.L	-	431	-24	6	-4.5	0.0051	2.6254
	STG.R	-	123	66	-19.5	6	0.0037	2.7436
	STG.R	-	144	60	-36	13.5	0.0129	2.2693
	ACG.L	-	260	-4.5	18	28.5	0.0071	2.502
	PreCG.L/PoCG.L	6	583	-39	-15	36	0.0004	3.4933
	PoCG.R/PreCG.R	4	429	21	-27	60	0.0007	3.3038
	SMA.R	-	201	7.5	-7.5	55.5	0.0007	3.3107
	PCL.L	6	217	-4.5	-27	60	0.0028	2.8415

Table S5: The best discriminating brain regions of GM for PD based on four algorithms.

SPEC	RELIEFF	RFE	STABLASSO
PCUN.R	PCUN.R	PCUN.R	PCUN.R
PCUN.L	PCUN.L	PCUN.L	—
CAU.R	CAU.R	CAU.R	CAU.R
CAU.L	CAU.L	—	—
STG.R	STG.R	STG.R	STG.R
STG.L	STG.L	—	—
MFG.R	MFG.R	MFG.R	MFG.R
MFG.L	MFG.L	MFG.L	—
MTG.R	MTG.R	MTG.R	MTG.R
MOG.R	MOG.R	MOG.R	MOG.R
MOG.L	MOG.L	MOG.L	MOG.L
INS.R	INS.R	INS.R	INS.R
INS.L	INS.L	INS.L	—
SFGdor.R	SFGdor.R	—	SFGdor.R
SFGdor.L	SFGdor.L	SFGdor.L	—
ORBinf.R	ORBinf.R	ORBinf.R	ORBinf.R
ORBinf.L	ORBinf.L	—	—
IPL.R	IPL.R	—	IPL.R
IPL.L	IPL.L	IPL.L	—
ANG.R	ANG.R	ANG.R	ANG.R
DCG.R	DCG.R	DCG.R	—
DCG.L	DCG.L	—	—
SFGmed.R	SFGmed.R	SFGmed.R	—
SFGmed.L	SFGmed.L	SFGmed.L	—
ORBmid.R	ORBmid.R	ORBmid.R	—
ORBmid.L	ORBmid.L	—	—
SMA.R	SMA.R	SMA.R	—
SMA.L	—	SMA.L	—
PoCG.L	PoCG.L	PoCG.L	—
ROL.L	ROL.L	ROL.L	—
PCG.R	PCG.R	—	—
PCG.L	PCG.L	—	—
ORBsup.R	ORBsup.R	—	—
ORBsup.L	ORBsup.L	—	—
REC.R	REC.R	—	—
REC.L	REC.L	—	—

—	—	PreCG	—
PreCG.L	—	PreCG.L	—
ACG.R	ACG.R	—	—
—	—	—	—
SMG.L	—	SMG.L	—
	PUT.R	—	—
PUT.L	PUT.L	—	—
PCL.L	—	PCL.L	—
ITG.R	—	—	ITG.R
OLF.L	OLF.L	—	—
IFGoperc.L	—	—	—

Table S6. The best discriminating brain regions of WM for PD based on four algorithms.

SPEC	RELIEFF	RFE	STABLASSO
—	PoCG.R	PoCG.R	PoCG.R
PoCG.L	PoCG.L	PoCG.L	—
ORBmid.R	ORBmid.R	ORBmid.R	ORBmid.R
ORBmid.L	ORBmid.L	ORBmid.L	—
SFGdor.R	SFGdor.R	SFGdor.R	SFGdor.R
IOG.R	IOG.R	IOG.R	IOG.R
LING.R	LING.R	LING.R	LING.R
ACG.R	ACG.R	—	—
ACG.L	ACG.L	ACG.L	—
PreCG.L	PreCG.L	PreCG.L	—
PCL.L	PCL.L	PCL.L	—
PUT.R	—	PUT.R	PUT.R
PUT.L	—	PUT.L	—
MOG.L	—	MOG.L	MOG.L
MFG.R	—	MFG.R	MFG.R
—	ORBinf.R	ORBinf.R	ORBinf.R
—	SMA.R	SMA.R	SMA.R
—	—	—	SMA.L
SMG.L	SMG.L	—	—
SFGmed.R	SFGmed.R	—	—
STG.R	—	—	STG.R
—	ORBsup.R	ORBsup.R	—
—	ORBsup.L	—	—
—	FFG.R	—	FFG.R
—	PCUN.R	—	PCUN.R
—	—	CAL.R	CAL.R
PCG.L	—-	—	—
THA.L	—-	—	—
—	REC.R	—	—
—	DCG.R	—	—
—	—	—	MTG.R

Table S7. Stability evaluation of selected ROIs in GM and WM tissues by the proposed methods.

GM	Stability score	WM	Stability score
PCUN	1	PCUN	0.5
STG	1	STG	0.5
MFG	1	MFG	0.75
MTG	1	MTG	0.25
MOG	1	MOG	0.75
ORBinf	1	ORBinf	0.75
PoCG	0.75	PoCG	1
ORBmid	0.75	ORBmid	1
DCG	0.75	DCG	0.25
SFGdor	0.75	SFGdor	1
SFGmed	0.75	SFGmed	0.5
SMA	0.75	SMA	0.5
ORBsup	0.5	ORBsup	0.5
PCG	0.5	PCG	0.25
REC	0.5	REC	0.25
PreCG	0.5	PreCG	0.75
ACG	0.5	ACG	0.75
SMG	0.5	SMG	0.5
PUT	0.5	PUT	0.75
PCL	0.5	PCL	0.75
CAU	1	—	
INS	1	—	
IPL	1	—	
ANG	1	—	
ROL	0.75	—	
OLF	0.5	—	
IFGopere	0.25	—	
—		LING	1
—		IOG	1
—		CAL	0.5
—		THA	0.25
—		FFG	0.25

*Stability score is defined as the frequency of brain regions identified by the four machine learning methods. The higher the stability score, the more probability the brain region to be the biomarkers of PD.

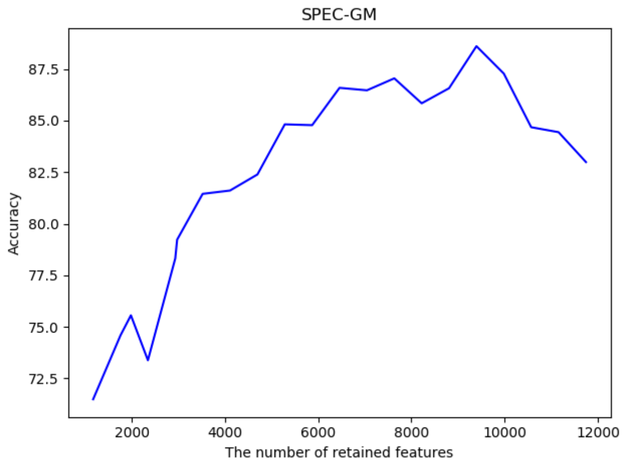

(1a) SPEC

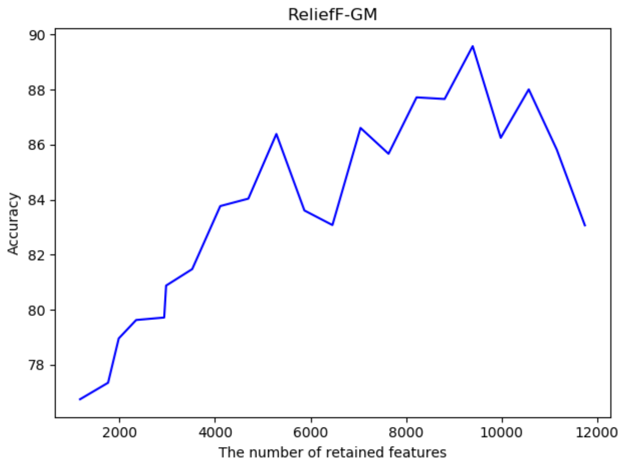

(1b) ReliefF

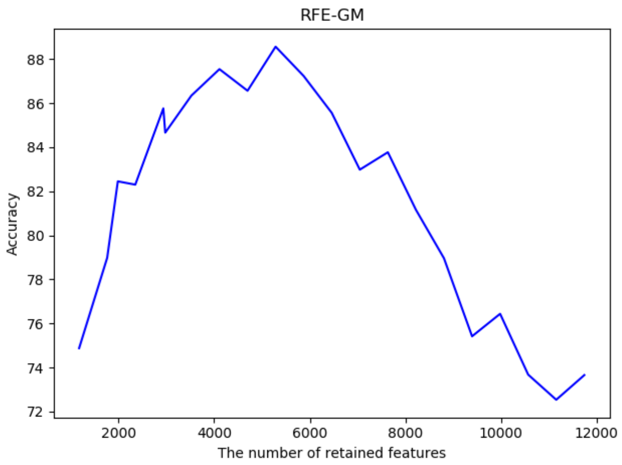

(1c) RFE

Figure S1: Feature-accuracy curves of different methods in GM modal.

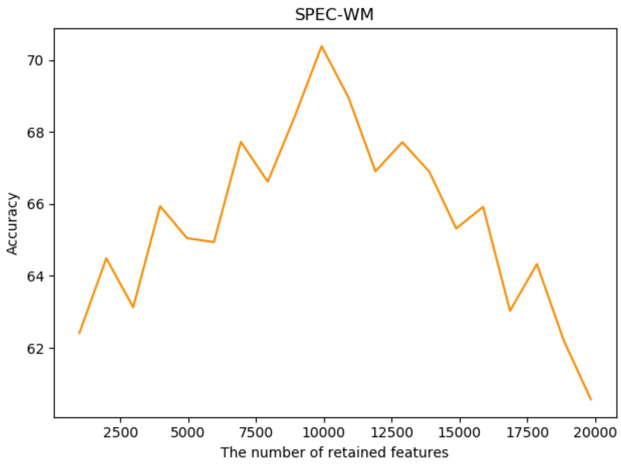

(2d) SPEC

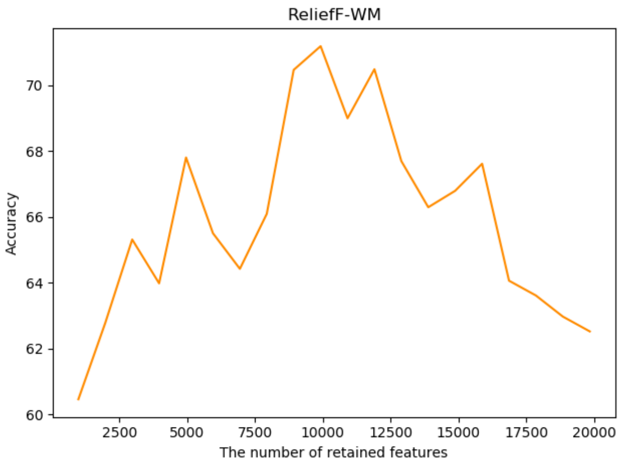

(2e) ReliefF

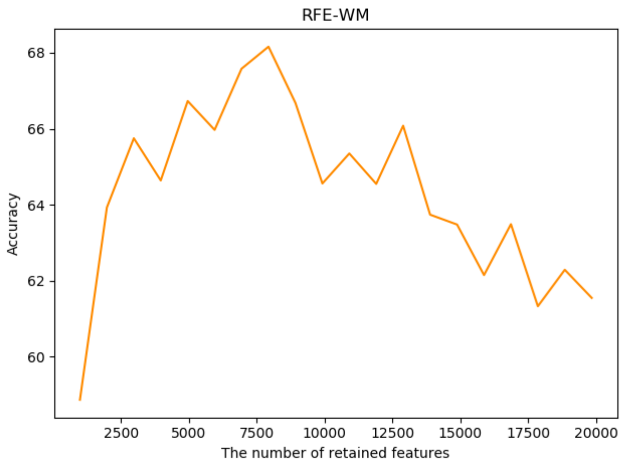

(2f) RFE

Figure S2: Feature-accuracy curves of different methods in WM modal.
